# An Evaluation of the Costs and Benefits of the New Psychological Support Service for Patients On Long-Acting Injectable Buprenorphine

**DOI:** 10.1192/bjo.2024.493

**Published:** 2024-08-01

**Authors:** Jan Melichar, Lucie James, Rosemary Greenwood

**Affiliations:** 1 Cardiff & Vale UHB, Cardiff, United Kingdom; 2 Aneurin Bevan UHB, Newport, United Kingdom; 3 University of Bath, Bath, United Kingdom; 4 University of South Wales, Newport, United Kingdom; 5 Cardiff University, Cardiff, United Kingdom; 6 UBHW, Bristol, United Kingdom; 7 Bristol University, Bristol, United Kingdom

## Abstract

**Aims:**

Long-acting injectable buprenorphine (LAIB) is an opiate substitution therapy which controls cravings and other symptoms for at least 28 days. Using this medication eliminates daily visits to the pharmacy, the risk of deviation or overdose from OST and death from overdosing with opiates. Many of those on LAIB return to the lives they left before they became addicted to opiates, but some require additional support from a new bespoke psychological service.

The new service with funds for two years opened in March 2023. An evaluation of the service is required before funding ends to ensure renewal. Here we discuss the costs, benefits and assumptions we have used to demonstrate cost effectiveness.

**Methods:**

Patients referred to the service are asked questionnaires and clinical outcomes at the start and end of the programme. We have used the EQ5D throughout as from this we can calculate the QALYs that NICE uses to value cost effectiveness. We have extrapolated that the benefit will last 4 years based on the COBALT study (Wiles 2016). We will be following the patients up at 12 months to test this but have no funds to follow up to 4 years. We have assumed that patients would have remained the same without this therapy.

**Results:**

The service has a budget of £23,812 a month and 5.17 patients a month (n = 31 July to December 2023) completed treatment. The average gain in EQ5D utility score per patient (n = 33) at discharge was 0.234 [0.140, 0.328]. If the quality-of-life score (EQ5D) at discharge is maintained in the same way as that for CBT in the COBALT study, the total number of QALYs gained from referral to 4 years would be 0.876. The cost-effectiveness ratio is therefore £5,261 per QALY gain. If the assumptions are correct and the patients retain this benefit this could be easily offset by savings elsewhere that have not been estimated in this evaluation.

**Conclusion:**

Our analysis shows we are cost effective, but we may be cost neutral due to the potential savings accrued due to less substance misuse – currently costed at £58K per user per year (Home Office). The use of additional questionnaires for assessing NHS resource use or criminal activity could derail the success by overburdening participants. Evaluating roll out to other sites needs to be costed and proportional.

With thanks to all who have helped with an infectious enthusiasm – we may be cost-effective.

